# Subspecialisation in Emergency Radiology: Proposal for a harmonised European curriculum

**DOI:** 10.3205/zma001138

**Published:** 2017-11-15

**Authors:** M. G. Wagner, M. R. Fischer, M. Scaglione, U. Linsenmaier, G. Schueller, F. H. Berger, E. Dick, R. Basilico, M. Stajgis, C. Calli, S. Vaidya, Stefan Wirth

**Affiliations:** 1 University Hospital, LMU Munich, Department of Radiology, Munich, Germany; 2 University Hospital, LMU Munich, Department of Didactics and Educational Research in Medicine, Munich, Germany; 3 Pineta Grande Medical Center, Department of Imaging, Castel Volturno, Italy; 4 Dartford & Gravesham NHS Trust, Darford, United Kingdom; 5 Helios Klinikum München, Department of Radiology, Munich, Germany; 6 Teleradiology Centre, Zurich, Switzerland; 7 VU Medical Centre, Amsterdam, The Netherlands; 8 Imperial College NHS Trust, St. Mary's Campus, London, United Kingdom; 9 Ospedale SS Annunziata, Chieti, Italy; 10 Gabinet Lekarski, Poznan, Poland; 11 Ege University Medical Faculty, Dept. of Radiology, Neuroradiology Section, Bornova Izmir, Turkey; 12 Barts Health NHS Trust, Royal London Hospital, London, United Kingdom

**Keywords:** Curriculum, Education, Medical, Radiology, Emergency Medicine, Quality Improvement

## Abstract

**Introduction:** Radiology plays a crucial role in the emergency care setting by delivering early and precise diagnoses under pressure of time, right at the beginning of patient treatment. Although there is a need for postgraduate education in emergency radiology, most of the national bodies responsible do not offer it in a uniform fashion and a general proof of qualification is missing in Europe. Therefore, the European Society of Radiology (ESR) has founded the (Sub-)Society of Emergency Radiology (ESER), prompting them to develop a European curriculum. This trend, which is currently also encouraged in many other non-radiological specialties which demand the highest professional qualifications, often lacks expertise in medical education.

**Goals: **The goal of this article is the general description of the curricular planning process for a European postgraduate subspecialisation programme, using the example of Emergency Radiology (European Diploma in Emergency Radiology, EDER), including the utilisation of TOOLS and recommendations derived from comparable projects.

**Project description: **The project was divided into partial steps: the timeline displayed in a GANTT chart, and tasks and responsibilities assigned in a RASCI matrix. The curriculum was iteratively developed using the KERN approach and steps were prioritised using the PARETO principle. Furthermore, the following TOOLS were used: limitations and needs assessment, SWOT analysis, formulating learning objectives and categorising them after MILLER and SCLO, and using BLOOM’s taxonomy for cognitive learning objectives and operationalising them according to MAGER. Psychomotoric and affective learning objectives were assigned to CANMEDS roles, grouped by topic using CLUSTERING, and then mapped by MATRIX analysis to appropriate learning and evaluation methods. Striving for continuous improvement, the curriculum was finally embedded in curricular quality management.

**Results: **The standardisation of the EDER access, considering the different national conditions, the minimisation of European learners’ attendance phases, restricting expenses by best possible use of existing structures, respecting the requirements and retaining the support of the European umbrella society ESR, finishing the project by a specific deadline and the demands of continuous improvement were particular challenges. A curriculum with the eligibility of five years’ speciality training in general radiology has evolved on schedule. The subspeciality training lasts at least one year and is divided into webinars, workshops during congresses (e.g. the annual ESR and ESER congresses) and one year practical training at the individual learner’s corresponding local hospitals, which adhere to a catalogue of requirements, comparable to national educational policies. The curriculum is completed by passing a written and oral exam (diploma) and re-accreditation every five years.

**Conclusions: **Despite complex requirements, the TOOLS utilised allowed an almost seamless, resource-minimised, professional, location-independent distributed development of a European subspeciality curriculum within one year. The definitive implementation is still due. If any deviations from the draft presented should become necessary in the future, the embedment in the curricular quality management will lead to a redirection in the right way and, furthermore, secure a continuous improvement in the best way possible.

## Zusammenfassung

**Einleitung:** In der Notfallversorgung nimmt die Radiologie durch frühe und präzise Diagnosen unter hohem Zeitdruck bereits zu Beginn der Patientenbehandlung eine zentrale Rolle ein. Obwohl ein eigener Weiterbildungsbedarf in Notfallradiologie gegeben ist, bieten die zuständigen nationalen Gremien dies meist gar nicht oder aber uneinheitlich an und es fehlt in Europa grundsätzlich ein Qualifikationsnachweis. Daher hat die Europäische Gesellschaft für Radiologie (ESR) die Subgesellschaft der Europäischen Notfallradiologie (ESER) gegründet und diese zur Entwicklung eines europäischen Curriculums aufgefordert. Dies ist eine Entwicklung, die derzeit zwar in vielen weiteren, auch nicht-radiologischen, Spezialisierungsbereichen vergleichbar motiviert wird, hierfür in aller Regel trotz höchster Fachqualifikation allenfalls wenig medizindidaktische Expertise zur Verfügung steht.

**Zielsetzung: **Ziel der Arbeit ist die grundsätzliche Beschreibung der Curriculumsplanung für eine postgraduierte europäische Schwerpunktspezialisierung am Beispiel der Notfallradiologie (European Diploma in Emergency Radiology, EDER) einschließlich der Angabe verwendeter TOOLS und der Ableitung von Empfehlungen für vergleichbare Projekte.

**Projektbeschreibung: **Das Projekt wurde in Teilschritte zerlegt, der Zeitablauf in einem GANTT-Chart festgehalten und Aufgaben sowie Zuständigkeiten mittels RASCI-Matrix abgebildet. Das Curriculum wurde unter PARETO-Priorisierung in den Schritten nach KERN iterativ unter Verwendung folgender Tools entwickelt: Limitations-, Bedarfs- und SWOT-Analysen, Formulierung der Lernziele und Kategorisierung nach MILLER und SCLO, Taxonomierung kognitiver Lernziele nach BLOOM und Operationalisierung nach MAGER, Zuordnung von CANMEDS-Rollen zu psychomotorischen/affektiven Lernzielen. Diese wurden mittels CLUSTERING zu thematischen Gruppen zusammengefasst und anhand von MATRIX-Analysen die geeignetsten Veranstaltungsformen, lernpsychologischen Methoden und Evaluationsarten zugeordnet. Mit dem Bestreben einer kontinuierlichen Verbesserung erfolgte abschließend die Verankerung in einem curricularen Qualitätsmanagement.

**Ergebnisse: **Besondere Herausforderungen waren die Vereinheitlichung des EDER-Zugangs bei sehr unterschiedlichen nationalen Voraussetzungen, die Minimierung von Präsenzphasen aufgrund des europäischen Teilnehmerkreises, eine Aufwandsminimierung durch bestmögliche Nutzung existierender Strukturen, die Berücksichtigung der Vorgaben des Europäischen Dachverbands ESR und Erhalt von deren Support, ein vorgegebenes Projektende zu gegebenem Stichtag sowie die Anforderung kontinuierlicher Verbesserung. Termingerecht entstand ein Curriculum, welches eine fünfjährige Weiterbildung in allgemeiner Radiologie als Zugang voraussetzt. Die mindestens einjährige Spezialisierung gliedert sich in Webinare, Präsenzworkshops während Kongressen (wie z.B. der ESR- und ESER-Jahreskongresse) sowie eine einjährige Weiterbildung an der jeweils lokalen klinischen Heimateinrichtung der Lernenden, welche sich nationalen Weiterbildungsordnungen vergleichbar zur Erfüllung eines speziellen Anforderungskatalogs verpflichtet. Den Abschluss des Curriculums bildet eine schriftliche und strukturierte mündliche Prüfung (Diplom) mit einer Re-Akkreditierung alle fünf Jahre.

**Schlussfolgerung: **Die verwendeten Tools erlaubten trotz komplexer Vorgaben innerhalb eines Jahres eine nahezu reibungslose, ressourcenminimierte, professionelle und ortsunabhängig verteilte Entwicklung eines europäischen Schwerpunkt-Weiterbildungs-Curriculums. Die konkrete Umsetzung steht noch aus. Sollten hierbei Abweichungen von dem präsentierten Startvorschlag notwendig werden, so verspricht die gegebene Verankerung in ein curriculares Qualitätsmanagement dies zumindest zeitversetzt auffangen zu können und auch darüber hinaus eine kontinuierliche Verbesserung bestmöglich zu sichern.

## List of abbreviations

- CBL = Case-based learning
- ECR = European Congress of Radiology
- EDER = European Diploma in Emergency Radiology
- EDiR = European Diploma in Radiology
- ESR = European Society of Radiology
- ESER = European Society of Emergency Radiology
- ETAP = European Training Assessment Programme
- ETC = European Training Curriculum
- L = Lecture
- LO = Learning objectives
- MC = Multiple choice
- Mini-CEX = Mini-clinical evaluation exercise
- MSF = Multi-source feedback
- NKLM = Nationaler Kompetenzbasierter Lernzielkatalog Medizin
- OSCE = Objective structured clinical examination
- PBL = Problem-based learning
- PDCA = Plan–do–check–act
- QM = Quality management
- RASCI = Responsible, Accountable, Support, Consulted, Informed
- SCLO = Swiss Catalogue of Learning Objectives
- SOE = Structured oral examination
- SWOT = Strengths, Weaknesses, Opportunities, Threats
- UEMS = European Union of Medical Specialists
- WFME = World Federation for Medical Education

## 1. Introduction

Emergency care is a general and permanent essential element in health care, with a very high proportion of emergency patients (e.g. about 20 million out of 80 million inhabitants in Germany annually [1]) and a further increase in the number of patient contacts in emergency departments worldwide [2]. Radiology takes a central role in this key process by delivering early and precise diagnoses at the beginning of patient treatment, which is not only medically, but also economically important [3], [4], [5].

### 1.1. Framework

The European countries have different training structures, durations and contents due to socio-economic, cultural and organisational reasons. The European Society of Radiology (ESR) is a radiological speciality society with over 63,000 members and currently integrates, as a European umbrella society, 47 national societies and 16 European subspeciality societies. One of the latter is the European Society of Emergency Radiology (ESER), which was founded in October 2011 [6].

#### 1.2. Motivation

**Figure 1:**
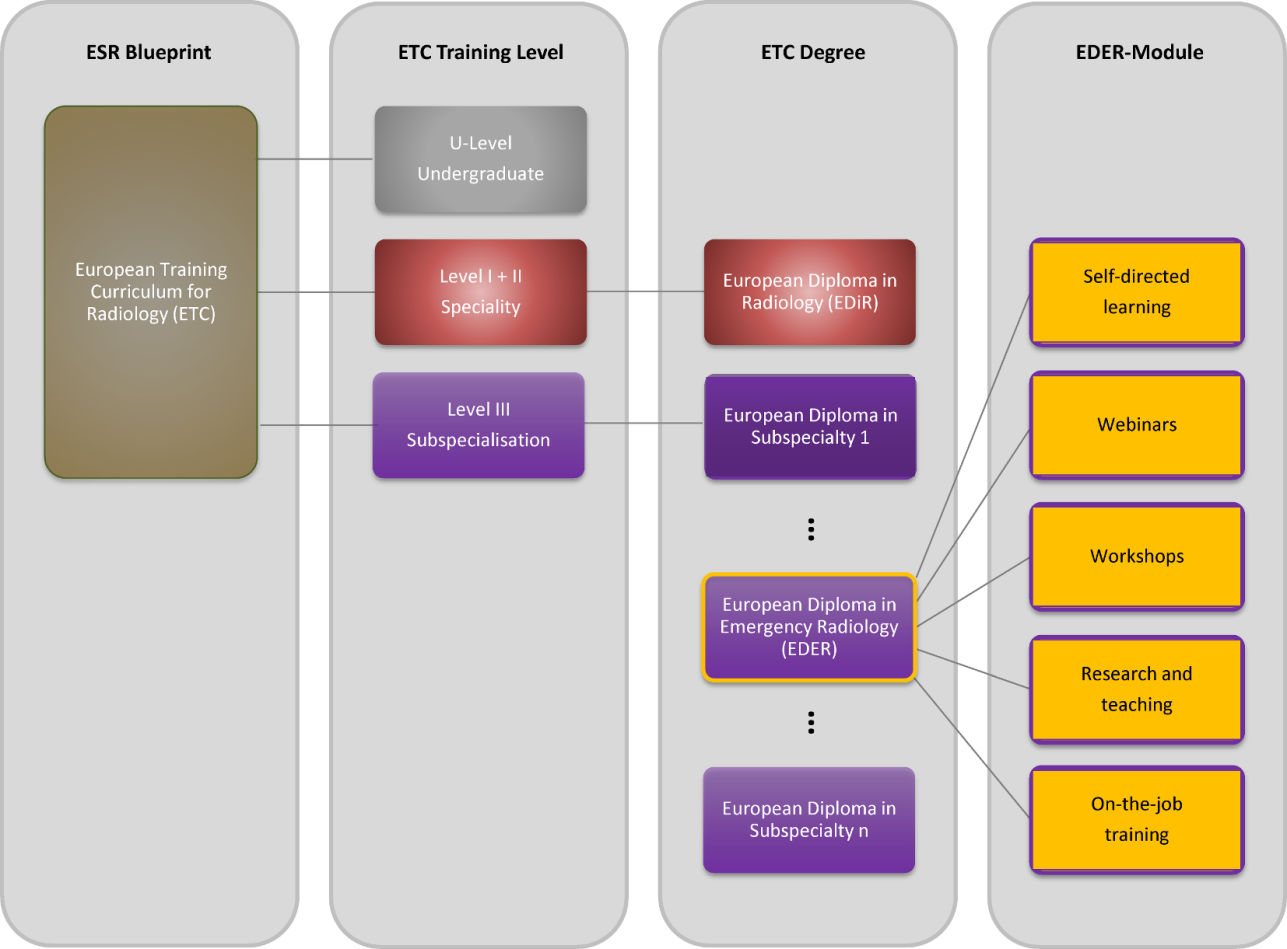
European Training Curriculum (ETC) of the European Society of Radiology (ESR). The ESR offers a training curriculum as a blueprint, which is filled with content by the European subspeciality societies. Level I and II are equivalent to speciality training and are graduated with the European Diploma in Radiology (EDiR). Subsequently, the subspeciality societies can offer a formal graduation of a LEVEL III subspeciality. Regarding the European Society of Emergency Radiology (ESER), this will be implemented with a European Diploma in Emergency Radiology (EDER). A comparable development can be observed in other national and supranational medical (sub-)speciality societies.

Despite the presence of a plausible need [7], few European countries have national speciality societies and no formal subspecialisation in emergency radiology is offered. Instead, it is considered sufficiently covered during the speciality training in general Radiology. In fact, a certain amount of emergency competence is present due to the frequency of emergencies, depending on the professional experiences and experts in emergencies who are present in countries without proof of qualification. In the US, a regulated acquisition of competencies and a corresponding certificate is given for this purpose [8], which also seems desirable for Europe. The ESR, whose guidelines are recommendations on a national level, has recognised those needs and engaged the ESER to not only develop a Curriculum for Subspecialization in Emergency Radiology, but also strongly encouraged them to offer their own European Diploma in Emergency Radiology (EDER). Consequently, the ESR provides a constantly updated, three-part European Training Curriculum (ETC; see Figure 1 (Fig. 1)) which can and should be used as a blueprint for basic radiological training (Level I as a common trunk, Level II as a specialist’s level) and optional subspecialisation training (Level III) [9], [10], [11]. 

The implementation of such projects by a specific, highly qualified panel of experts, who have no comparable expertise in medical education and no possibilities to acquire those competencies externally, is a particular challenge, in comparison to the classical situation of university teaching.

The challenges are expected to be comparable to the example of European emergency radiology described here [e.g. [12], [13], with an already present and probably continuously increasing demand for specific, supranational curricula throughout the whole sector of postgraduate medical education.

## 2. Goals

The goal of this article is the general description of the curricular planning process for a European postgraduate subspecialisation programme, using the example of emergency radiology (European Diploma in Emergency Radiology: EDER), including the utilisation of TOOLS and recommendations derived from comparable projects.

## 3. Project description

SW. as a member of the ESER Executive Board, was commissioned to develop a proposal for a curriculum for the subspecialisation in emergency radiology (see 1.2. and Level III in Figure 1 (Fig. 1)) which meets the formal ESR requirements of the ETC, highly probable finds acceptance at the various national societies and is attractive to candidates (learners). Because the ESR, as the umbrella society, has pre-financed the founding of the ESER, their fundamentally needed support requires the development and implementation of the curriculum to be cost efficient, since the financial situation is still tense, although decreasing.

### 3.1. Project start

Firstly, the ESER Board collected specific strengths, weaknesses, chances and threats using BRAINSTORMING and processed them by SWOT analysis [14] to identify special project demands and inevitable limitations. Consequently, the maximal amount of educational methods and minimal duration of training resulted. The goal of the project was defined, the timeline determined and the project was divided into subtasks which were displayed in a project plan [15], [16], [17], responsibilities were displayed in a RASCI matrix [18], [19], subtasks were distributed to the individual members under the responsibility of SW and the project was released (KICK-OFF).

#### 3.2. Project progress

The KERN model [20] for the development of medical curricula was used and the World Federation for Medical Education (WFME) “Global Standards for Quality Improvement – European Specification” [21] were met. A catalogue of learning objectives was developed and categorised by Knowledge, Skills and Competencies/Attitudes [10], adhering to the requirements given by the ESR. Additionally, the cognitive learning objectives were categorised, using the Revised BLOOM’s Taxonomy [22], and operationalised after MAGER [23]. Psychomotor and affective learning objectives were assigned to CANMEDS roles [24]. Finally, a CLUSTERING [25] of learning objectives into thematic groups, educational methods, and formative and summative evaluations was carried out and quality management (QM) was established to secure the flexibility and continuous improvement intended.

## 4. Results

The project was launched during the ESER board meeting in October 2015. SW was commissioned, using the results from Table 1 (Tab. 1) (left and centre), to develop a project plan within one month (GANTT chart [15], [16], [17], see Table 2 (Tab. 2), on top), including the assignment of responsibilities (RASCI matrix [18], [19], see Table 2 (Tab. 2), below), specify the SWOT analysis with concrete actions (see Table 1 (Tab. 1) below) and optimise it by circulation procedures with the board.

### 4.1. Important results and consequences of the preliminary work

- The ETC blueprint is subject to change and the curriculum development needs to be prioritised to adhere to the deadline.
- A strong relationship to the ESR is necessary to preserve their support and get cost neutral or discounted usage of their infrastructure.
- Some learning objectives can only be met in a decentralized way in an external institution; in most cases, this is the learner’s home institution/hospital.
- Other learning objectives will be distributed to decentralized self-learning, and central webinars and workshops at annual ESR and ESER congresses.
- Acknowledgment of extracurricular performance and expertise should be possible.
- The offering of EDER must not worsen the financial situation of ESER.

#### 4.2. Brief summary of the results of the KERN cycle

**Figure 2:**
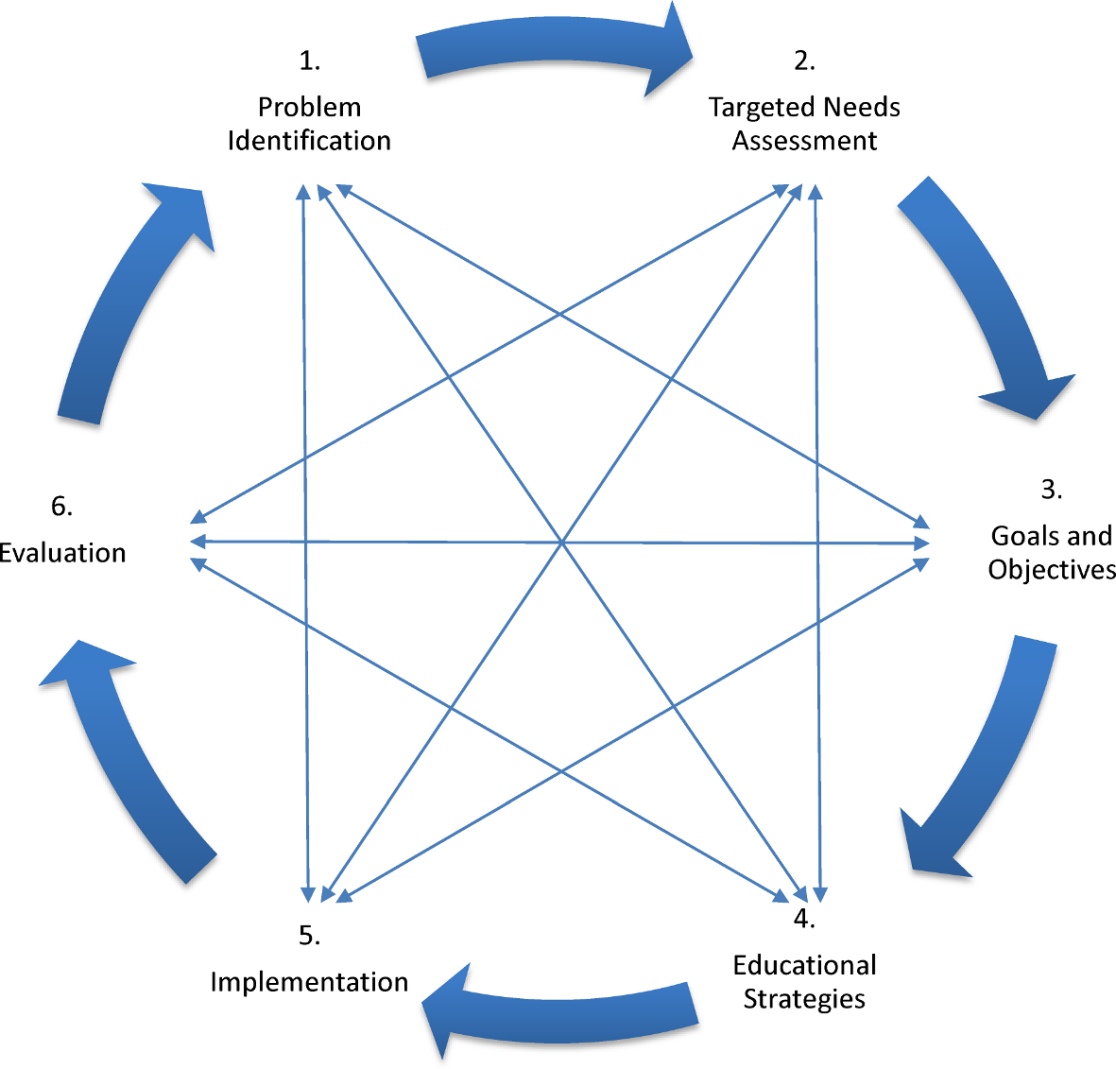
KERN cycle for medical curriculum development [17]. The KERN cycle presents a six-step approach of curriculum development for medical education (thick arrows). The thin arrows illustrate dependencies and consequences regarding all other steps. Consequently, it is advisable to reiterate the cycle stepwise, not only to get a better curriculum, but also to become flexible at reacting to immutable alterations. This corresponds ultimately with the basic principles of quality management and the endeavour of continuous improvement. (LO = learning objective)

The basic steps presented particularly respected the PARETO principle [26]. A “perfect” curriculum was waived to meet the deadline given, but continuous improvement was embedded. This means that an iterative process of the KERN cycle (see Figure 2 (Fig. 2)) is used within the framework of curricular QM (see 4.3).

##### 4.2.1. Step 1: Problem Identification and General Needs Assessment

*Health care problem and current approach:* Also see “1. Introduction” and especially “1.2 Motivation”. Emergency radiology is, in principle, integrated into national speciality training. The duration and contents are, nevertheless, not standardised, and the possibility of displaying expertise at a broader level is only possible by showing engagement at the few national societies for emergency radiology. In most cases, a minimal duration of training in the speciality of general radiology, in combination with some procedures at a given modality, is required instead, which is sometimes divided by anatomic regions. In this case, emergencies are contained implicitly, but proof is not necessary. Although there are general recommendations by the WFME for postgraduate training in Europe, they are not implemented for Emergency Radiology [21].

*Ideal approach: *Graduates should receive a certificate which proves their achievement of competencies in Emergency Radiology in a regulated and standardised manner. The curriculum should be equally appealing for learners and teachers, maintain support from the ESR, promote new ESER memberships and, at least, not worsen the financial situation of ESER. The embedment in curricular QM should, furthermore, obtain the flexibility and secure continuous improvement wanted.

##### 4.2.2. Step 2: Targeted Needs Assessment

*Targeted Learners:* Learners with a special interest in Emergency Radiology, at least five years of speciality training in general radiology and a good command of the English language, which is sufficient enough not to hinder their success in lectures and exams.

*Needs Assessment of Learners: *No European-wide survey has been carried out due to limited resources and time constraints. Instead, the last ESER general assembly was used to be as representative as possible to gather informal opinions. On this occasion, the possible learners attached value to a high number of lectures through the Internet or at the home institution, the proposal of collective, centralised lectures, preferably at the congresses visited, a compact range of highly practically oriented courses and a high acquisition of competencies.

*Needs Assessment of the Learning Environment: *The ESR offers facilities, personnel and equipment (e.g. server, computers, monitors, software, projectors, whiteboards) to carry out cost-effective webinars, workshops and exams with a reduced organisational effort from the annual congresses. A sufficient web-based learning, teaching and administration platform for exam questions (i.e. the Item Management System [27]) is not currently available and should ideally be acquired, installed and administered by the ESR. 

##### 4.2.3. Step 3: Goals and Objectives 

The overarching goal after completion of the programme is a high level of emergency radiological expertise. Consequently, knowledge, skills and competencies/attitudes are taught to guarantee the secure and effective handling in radiological diagnostics and therapy, according to current standards. The specific learning objectives were divided into Knowledge, Skills and Competencies/Attitudes, according to the ETC blueprint, and written down in the Level III Curriculum ([11], pp. 34-7).

*Cognitive: *The main goal is the acquisition of knowledge for the application of adequate radiological imaging and therapy in emergency situations using the appropriate modality.

*Psychomotor and affective: *The supervision of the correct execution and interpretation of radiological diagnostics according to current standards, motivation for individual improvement, promotion of a positive team atmosphere, development and execution of measures for quality assurance and development, demonstration of good communications and teamwork, continuous medical education and the effective dissemination of medical knowledge are all paramount.

The specific learning objectives are too comprehensive for presentation on this occasion, but are listed as an overview on the ESR homepage [11] and should be made available in an actualised and more detailed version on the ESER homepage [28] in September 2017.

##### 4.2.4. Step 4: Educational methods (and content)

The number of educational methods was restricted in favour of an assured handling of the contents.

*Webinars: *The topics were aggregated into nine groups, mostly anatomically, by using a CLUSTERING [25] of learning objectives:

1. head/brain;
2. spine, spinal cord and peripheral nerves;
3. general fractures;
4. Heart and vessels;
5. angio-intervention;
6. thorax: non-traumatic, including intervention;
7. Adom not-traumatic; including intervention;
8. musculoskeletal; and
9. polytrauma.

These groups were outside the borders of the cluster:

1. special patient groups, such as children, pregnant women, elderly and tumour patients, and
2. miscellaneous, such as drugs, quality assurance, legal bases and radiation protection.

All webinars are bracketed by a preparation and follow-up phase. As an online offering, the preparation of about 30 minutes includes basic principles and repetition of prior knowledge from Level I and Level II for a better understanding of the webinar. The follow-up of about 60 minutes supplements the contents of the webinars with appropriate cases and leads to a deeper understanding.

**Figure 3:**
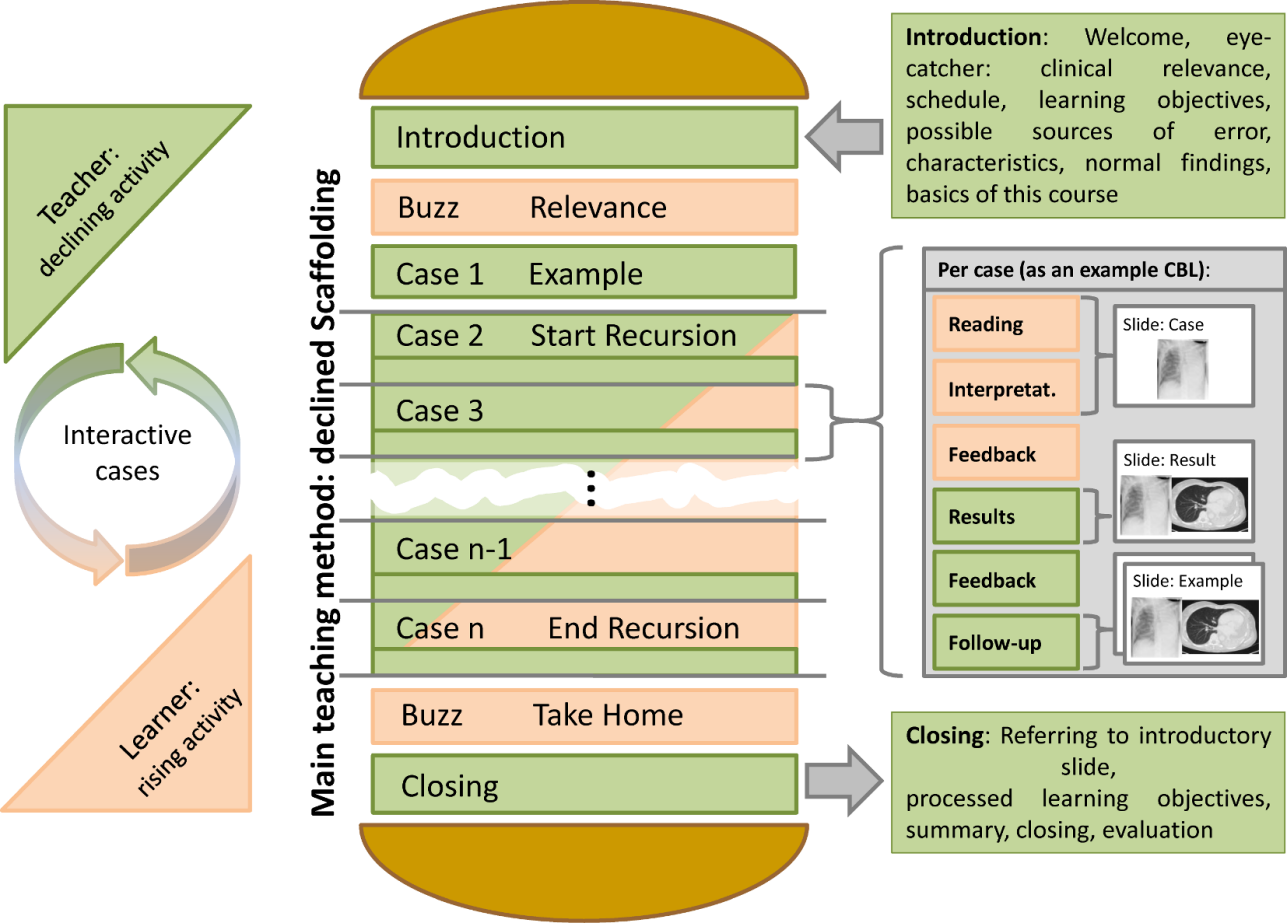
General didactic structure of “deceasing scaffolding in case-based sandwich technique”, as a 90-minute workshop, using the example of thoracic imaging of emergencies, (also see Tab. 3 and 4). The key teaching method is “case-based learning” (CBL) with “decreasing scaffolding” (progressing activity of learners with declining help of teachers) in a “sandwich technique” (interactive approach with changing activity level of learner and teacher). An important integrated element of learning psychology is Feedback by teachers and learners among a group. This is framed by Buzz groups at the beginning and end, with the objective of raising attention and sustainable dissemination of knowledge. The introduction is initially purely frontal and limited to a maximum of 20 minutes for reasons of learning psychology.

*Workshops: *The basic sequence of the workshops occurs with the changing activity levels of learners and teachers as a “decreasing scaffolding in a sandwich technique” [29] (see Figure 3 (Fig. 3), Table 3 (Tab. 3) and Table 4 (Tab. 4)). Emphasis is on case-based (CBL) [30] and problem-based learning (PBL) [31], [32], [33], [34], as well as FEEDBACK [35], [36]. CanMEDS roles [24] were prioritised by topics using MATRIX analysis [37]. Comparable with the webinars, all workshops are bracketed by a preparation and follow-up phase.

*Self-directed learning: *This serves as a complement to teaching cognitive learning objectives and takes place within the context of scientific work.

*Teaching hospitals:* In addition to psychomotor skills and competencies, almost all affective learning objectives need the environment of an acute care hospital. The implementation cannot be centralised and, therefore, needs to be conducted in an appropriate institution. Appropriate institutions need the ability to achieve the case numbers required, implement CBL and PBL as learning methods, and prove the presence of expertise in medical education and didactics [30], [31], [32], [33], [34].

##### 4.2.5. Step 5: Implementation

**Figure 4:**
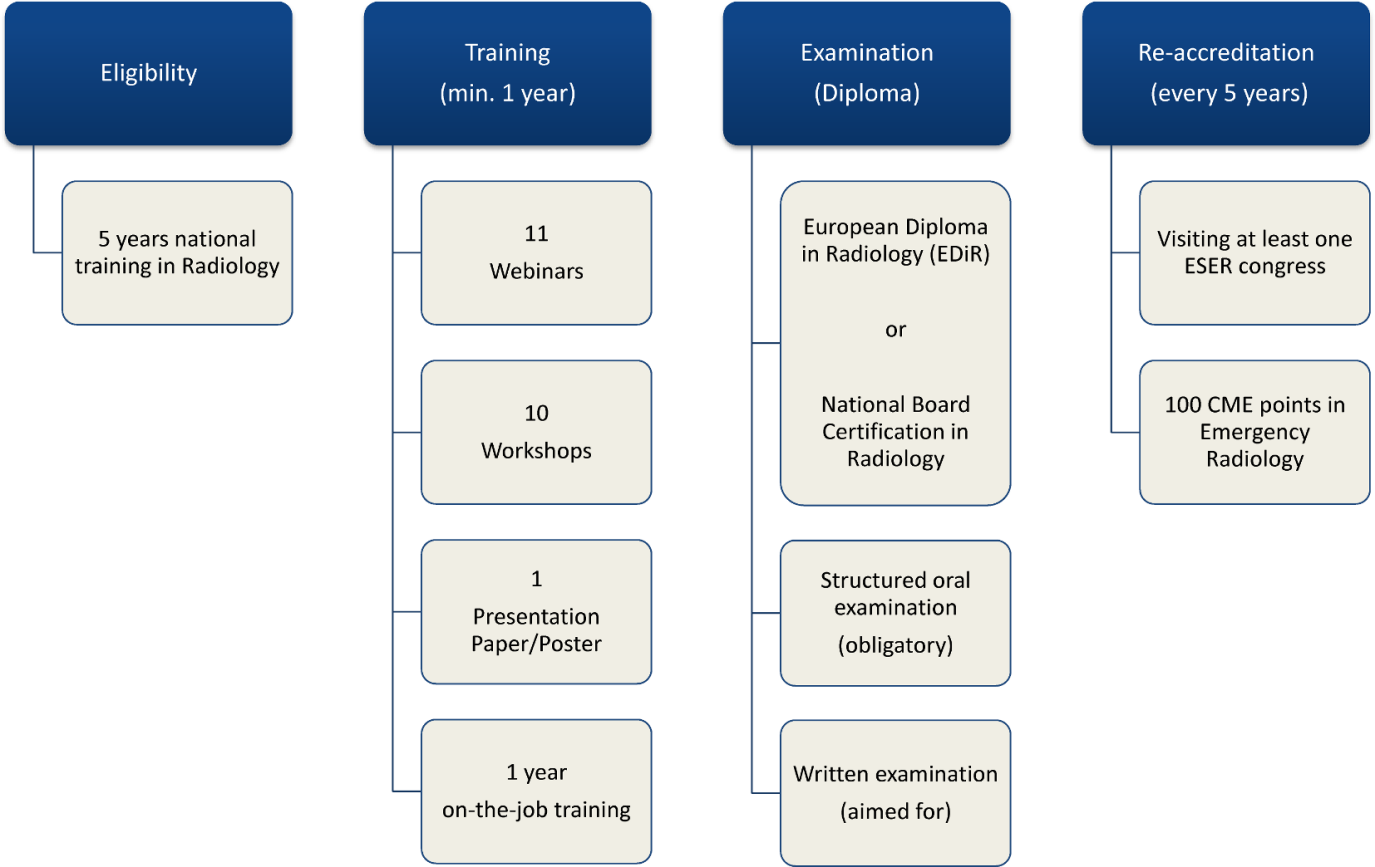
Resulting curriculum structure

The curriculum follows a modular fashion (see Figure 1 (Fig. 1) and Figure 4 (Fig. 4)) and will be introduced in the autumn of 2017. The minimum duration needed for the decentralized training necessary at a teaching hospital determines the minimum time of training and was determined, by the extent of the content, to be one year. Teachers will be schooled separately and the curricular QM is described in 4.3.

*Webinars:* Eleven webinars of 60 minutes each and up to an additional 30 minutes discussion will be offered in a monthly rhythm in the timespan of one year. The contents correspond with the nine topics described at 4.2.4 and the overarching topics: (10) special patient groups and (11) miscellaneous. The online preparation (30 min) and follow-up (60 min) use E-LEARNING [38], [39] and INVERTED-CLASSROOM concepts [40]. The webinar equipment is provided by the ESR and an electronic learning platform (i.e. Item Management System [41]) should ideally be acquired, installed, adjusted and administered together with the ESR. The ESR is currently beginning to offer learning modules online with the programme “Education on Demand” [https://cslide.ctimeetingtech.com/library/esr/homeearn]. This platform allows the webinars to be streamed independent of time after recording.

*Workshops*: Five workshops per day, each of 90 minutes, will take place at the annual ESR and ESER congresses. The ten workshops are grouped by topic, similar to the webinars 1 to 9, and topics 10 and 11 are summarised in a tenth workshop (see Figure 2 (Fig. 2), Table 3 (Tab. 3) and Table 4 (Tab. 4)). They especially integrate complex case situations, video examples and group practices regarding the CanMEDS roles [24]. The structure of the online preparations and follow-ups are comparable to the webinars. One objective structured clinical examination (OSCE) will be carried out on each workshop day. The necessary equipment will be provided by the congress organisers.

*Self-directed learning:* This involves predetermined obligatory and facultative literature, including ESER books, which currently cover topics (3) to (9), partially (2) and ungrouped contents.

Scientific work: Within the scope of the ‘Scholar’ role, an understanding of research for decision-making on a scientific basis is a prerequisite for the participants or needs to be self-taught. In this particular case, ESER does not offer a concrete proposal, but requests a proof of performance (see 4.2.6)

*Teaching hospitals: *In addition to all the psychomotor skills and competencies, almost all affective learning objectives need the environment of an acute care hospital, which is why the teaching of those learning objectives needs to be transferred to decentralized institutions. Consequently, learning objectives were separately categorised, grouped and listed, and a relevant logbook conceptualised. The institutions will be bound by contract to fulfil a given framework of content and time: lectures, a catalogue of autonomously conducted diagnostics and interpretations, intermediate exams, feedback discussions, attitudes and on-call duties.

##### 4.2.6. Step 6: Evaluation

The evaluation takes place on four levels regarding the learners and the programme; training at the teaching hospital will be evaluated semi-annually. Webinars/workshops will be evaluated formatively after each lecture (L) and summatively at the end of the programme. All formative evaluations use the same Likert scale from 1-6 (strongly agree – strongly disagree) and contain free text questions (“What was good?” and “What could be better?”).

Self-directed learning: The summative evaluation of the obligatory literature occurs during the final exams.

*Webinars: *Preparation and follow-up each include five multiple choice questions (MC) chosen randomly from a pool of questions. The lecture itself will be evaluated formally. Questions are, for example, “The knowledge offered was new to me?”, “The number of contents was appropriate for the duration of the lecture?” and questions about the quality of the teacher. Proof of performance for the 11 webinars are the MC tests passed and certificates of attendance.

*Workshops:* Questions in the KEY-FEATURE format [42] will be used accounting for 50% of the possible score, similar to the webinars. Proof of performance are two OSCEs passed [43], [44], exams in each workshops and certificates of attendance.

*Scientific work:* Proof of performance is the individual presentation of a paper or poster about an emergency radiological topic at an ESER-approved congress, or the acceptance as a first or last author of a manuscript for review in a indexed, peer-reviewed journal. Alternatively, three co-authored manuscripts will be accepted. This scientific work may be carried before the beginning of the programme and emergency radiological relevance will be verified by ESER.

*Teaching hospital:* Eleven radiology-adapted mini-clinical evaluation exercises (Mini-CEX) [45], [46] and one structured FEEDBACK discussion with a mentor must be carried out and positively recorded in the logbook during practical training, according to the topics and cycle of the webinars. On the first, sixth and twelfth month, a multi-source feedback (MSF) [47], [48] (respectively one attending radiologist, resident, technician and patient feedback) results in the achievement of the corresponding competence level in the seven CanMEDS roles [24] from 1-6 (full – no competence). Furthermore, an evaluation regarding the institution will be carried, similar to that described above at “webinars” and “workshops”. The completed logbook with the achievement of all learning goals, signed by the chair of the institution, provides proof of performance.

*Final exam: *Admission follows the submission of all proofs of performance named above. An application for the recognition of alternative proofs of performance can be filed. A successful certificate of training for national speciality training or a European Diploma in Radiology (EDiR) must be presented before admission. The summative evaluation will be provided, at least, during each ESR and ESER congress and includes a structured oral examination (SOE) [49] for evaluation of psychomotor and affective learning objectives, 20 MC questions [50] for evaluation of knowledge (50% of the score) and questions about five cases in KEY-FEATURE format [42] for evaluation of procedural knowledge. Both exams must be passed on the same day to receive the diploma.

#### 4.3. Curricular quality management

The limited personal and financial resources require a particular focus on a strong QM. Consequently, a QM commissioner with curricular and QM competencies was nominated by the ESER board. The remaining board members are responsible for partial aspects and the development of process descriptions and operating procedures. The Plan-do-check-act or Deming cycle (PDCA) [51] will be undergone annually with the goal of continuous improvement. During the ongoing operations, customer satisfaction analysis, in addition to the monitoring of key figures, complaint and risk management, will also take place, intending to optimise processes and the strategic planning actively, while regarding the initial vision and mission. A QM conference is an integral part of the annual ESER general assembly at the European Congress of Radiology (ECR), and a mid-term report follows at the annual ESER board meeting.

The following key figures are of particular concern:

- the number and distribution of learners, teachers and certified teaching hospitals,
- the success of the learners during the programme and at examinations and ratings of teachers and learners,
- the number and quality of exam questions [52], and
- the curricular profit and loss account.

## 5. Discussion

The curriculum development presented, with the dedicated time frame, limited resources, uniqueness of its conditions and goal of achieving available quality standards, complies with the definition of a project according to the German Institute for Standardisation (DIN), the Project Management Institute (PMI) and the International Project Management Association (IPMA) [53], [54], [55]. As such, established TOOLS of project management were combined with those proven during curriculum development. The Pareto principle was adopted with the aim of feasibility and compliance with deadlines given. Hence, various limitations were accepted which are described further below. Nevertheless, under these circumstances, this curriculum proposal has an idealised form which might change during implementation if the initial experiences and data evaluations support that. Consequently, a conflict between limiting specifications, such as minimising resources or support of various national societies, and the broader goal of a high radiological expertise in emergency care evolved. Those aspects could be counteracted by the integration of a curricular QM, which is part of the continuous improvement process.

The approach at the beginning of the project was unusual and occurred because of internal considerations to increase the necessary, but not ensured, support of the ESER board. Therefore, e-mail communication was not considered to be expedient and instead, a private meeting, during the few in-person occasions was chosen. The initial BRAINSTORMING at the board meeting concerning strengths, weaknesses, opportunities and threats promoted, in addition to an early estimation regarding the feasibility of the project, the communication between curricular staff and revealed individual ideas. The following SWOT analysis [14] derived clear strategies, despite the complexity of the project, laid out a basis for discussion with the stakeholders and was considered to promote team spirit. We trace that to the active and immediate involvement of the staff, displaying of project potential and related problems, and work density. This again suggested a demand for commitment to active support, and what happened voluntarily and was written down during the protocol meeting would serve as a reliable statement. Furthermore, the consecutive creation of a project plan, with the established mapping using a GANTT chart [15], [16], [17], not only helped the visualisation, but also pointed out individual steps and pressure of time, leading to an increased willingness of board members to accept tasks. The RASCI matrix [18], [19] helped to overcome problems emerging from the physical distance between the curricular staff by defining clear responsibilities to specific tasks. ESER is a small society with only a few members (currently 117, coming from 29 countries, with 6 outside the EU). Regarding the teachers, the prospect of the acknowledgement of certain expertise to help pass the programme and obtain the examiner’s qualification was especially motivating, and to accept tasks during curriculum development by serving as teachers and examiners. The prospect of a possible improvement of the ESER finances due to gaining more members, a higher rate of congress visitors and using the existing infrastructure of an umbrella society was helpful to maintain the support of the ESR. Although we were pleased that several acute care hospitals contacted were generally willing to undergo a fee-based certification process and considered it supportive if candidates motivated local hospitals to do so, we postponed it for reasons of feasibility. Instead, the curriculum launched a catalogue of requirements defined by duration and content which must be fulfilled and signed by the director of the institution, comparable to the approach of national (sub-)speciality training. We aspire to the certification of such institutions within the framework of continuous improvement over the coming years and we started sending out questionnaires (which especially regard the improvement of a special needs analysis) to the directors and future learners. Since recognised experts among the active ESER members have a large interest in getting to know foreign acute care hospitals and their corresponding emergency radiology departments on-site by this means, we are confident that certifications will be possible in the future. We understand this and the motivation of learners as a “hidden curriculum” [56].

An early commitment to possible educational methods was helpful during the concrete implementation. Webinars, for instance, are already being held and only need to be restructured. It should also be easy to maintain a majority of the teachers currently active. Additionally, we chose the requirement to fulfil the learning objective in research and science since Seaburg et al. (2016) in a recently published study, found a significant association between the number of publications and ratings in clinical evaluations of speciality trainees [57], and we demand that participants must base their decisions on scientific facts.

Our first estimation of the curriculum development under the situation given, using the approach and TOOLS above, is subjectively positive. An objective opinion will be possible after the concrete implementation, arrival of the evaluation results and comparison with the target values. The possibility of an external assessment is present with the European Training Assessment Programme (ETAP), an initiative founded by the ESR Education Committee in 2001 and part of the European Union of Medical Specialists (UEMS) [58]. The ETAP analyses and rates, among other things, the structure, implementation, educational methods and materials, venues and outcome on-site, and gives final recommendations. A promotion portfolio can be created, together with the first summative evaluation results, to raise the support of stakeholders further.

### Limitations

The TOOLS presented and the general approach of curriculum development have already been published multiple times. The originality of this article lies in the condensing of information for the increasingly frequent case of comparable projects initiated by professional societies, particularly on a supranational level, often planned and implemented with the same challenges. This happens frequently with established specialist competencies, but without added comparable competencies in medical education and without the possibility of secondary consultations. The article presented should raise awareness and serve as a compendium for a professional approach.

The informal targeted needs analysis of the learners (Step 2 of the KERN cycle [20]), carried out during the ESER board meeting with ESER members, is a deficiency of the project. A preliminary, methodically perfect evaluation on a European level is time-, cost- and resource-intensive, without ensuring a better implementation and appreciation of the stakeholder. Since we focused on fast feasibility using the PARETO principle [26], the special needs analysis will be refined annually within the framework of continuous improvement. Finally, comparable restrictions apply to other aspects of the KERN cycle [20], especially to implementation and evaluation.

Every institution operating as a teaching hospital in the future, should be charged for certification by ESER, auditioned annually and recertified every three years. The ESER board will seek professional consultation to decide on the specific standards used. We have to proceed as is common at the German Medical Association (and others) for reasons of feasibility: A simple written confirmation about the fulfilment of performances/requirements catalogued is produced which, in principle, is not verifiable by ESER itself.

The summative evaluations at the end of the curriculum are limited by the time available at the annual congresses and the number of teachers and examiners. Should the number of candidates interested in EDER rise, as was the case with EDiR (less than 100 at the beginning of 2011 to more than 1000 candidates at the end of 2015), the SOE could reach its limitations fast. The European Board of Radiology, which conducts exams for EDiR, implemented a new assessment format successfully in 2016, which replaced the oral exam and simultaneously improved the assessment methodology [59]. In addition to 75 MC and 24 short case questions, a Clinically Oriented Reasoning Evaluation (CORE; 90 minutes) with ten cases on a DICOM viewer for imaging interpretation was used. In the case of a fast increase of EDER participants, the joint application of existing EDiR resources and the sharing of know-how could become very helpful.

## 6. Conclusion

The preparation of a European subspeciality qualification in Emergency Radiology has led to a curriculum development with the following limits and requirements which may apply to many other European curricula for postgraduate medical education:

- Limited time is confronted with the pressure to succeed
- People responsible do not always have a high expertise in medical education
- Eligibility needs to be unified, despite various national conditions
- Minimal synchronous attendance time for learners, teachers and people responsible
- No separate financial or personnel resources

We recommend a close co-operation with experts of medical education and QM for any comparable projects. The initial needs analysis with the determination on a subsequent framework, followed by a stepwise PARETO-prioritised development, protected by a QM for continuous improvement exists as a working model. From our perspective, the following project TOOLS were shown to be helpful: BRAINSTORMING, CHECKLISTS, SWOT analysis, GANTT chart, RASCI matrix, MATRIX analysis and CLUSTERING of topics. We suggest NKLM, Revised BLOOM’s Taxonomy and CANMEDS roles for categorising learning objectives. Webinars and workshops during speciality congresses seem beneficial as teaching formats. The inclusion of teaching hospitals is inevitable for practical training with real patients. We recommend the limitation of a few educational methods, since it is easier to mediate those to the teachers on a high level and this, consequently, yields better handling: keynote presentations and mainly SCAFFOLDING in SANDWICH technique, FBL, POL, FEEDBACK BUZZ groups and the extensive use of E-Learning for online preparation and follow-ups. We suggest SMP for oral and a mix of MC with KEY-FEATURE as assessment formats for written exams. 

By implementing special expertise and utilising the methods and TOOLS above, an almost seamless, resource-efficient, professional and location-independent development of a European subspeciality training curriculum succeeded, including the integration of a QM, within one year.

## Notes

Although the text was phrased gender neutrally in principle, the generic masculine form was used in some passages for reasons of simple legibility.

The project is not financially supported. ESER, as a society, has received funds from sponsors for congress events for an adequate value in the past. The total amount in the last three years was below 100,000 Euro and these funds had no influence on the manuscript presented here.

## Acknowledgement

Parts of this work originated within the framework of the dissertation project of Martin G. Wagner.

ESER thanks the ESER office in Vienna, represented by the deputy, Wolfgang Duchek, and the project secretary, Sabine Grab, Department of Radiology at the Ludwig-Maximilians-University in Munich, for their great support.

## Competing interests

The authors declare that they have no competing interests. 

## Figures and Tables

**Table 1:**
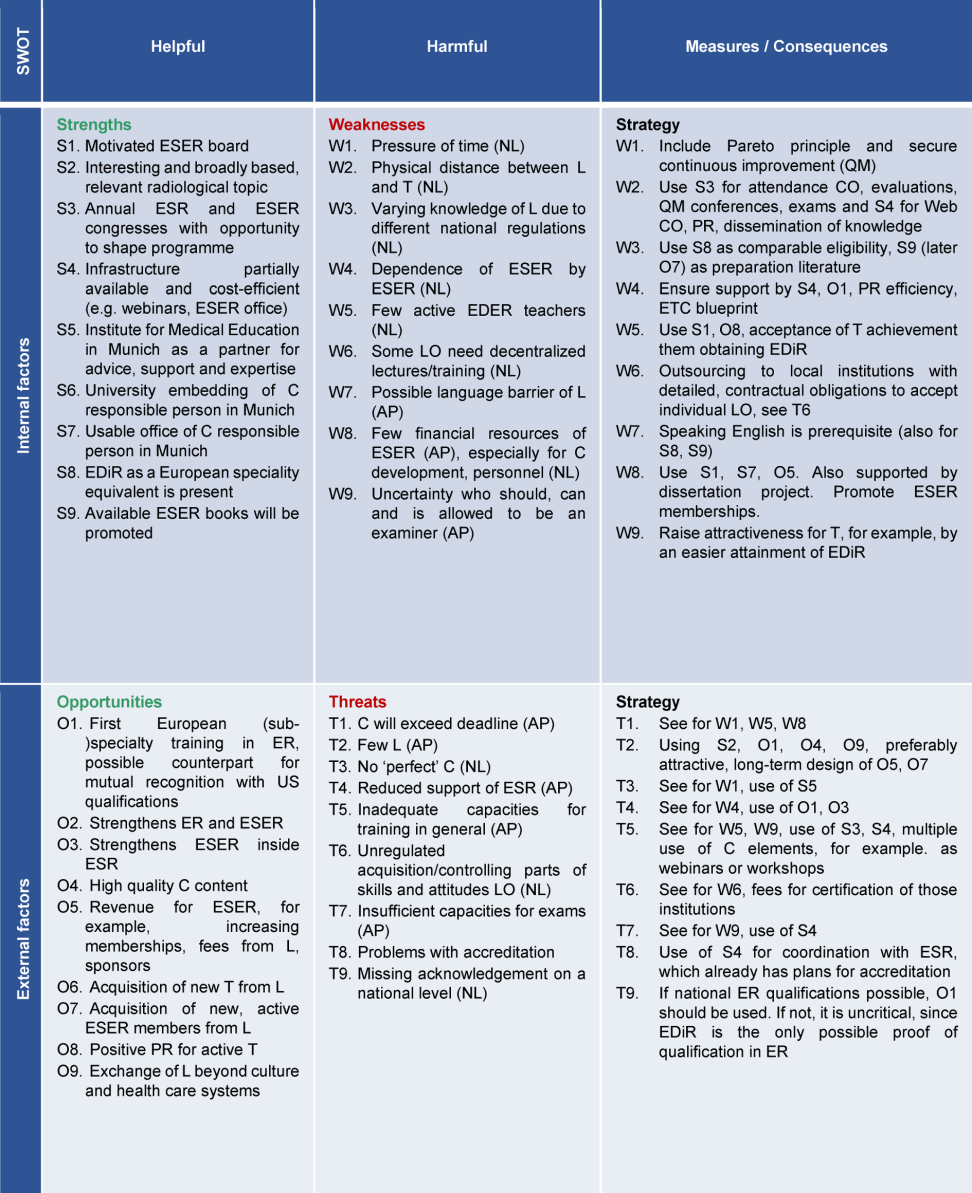
SWOT analysis of EDER project with strategic reappraisal (counter-measures for weaknesses/risks by using strengths/opportunities), each with a prioritised statement of the nine most important items. Abbreviations: AP = adjustable problem, C = curriculum, L = learner, T = teacher, curriculum developer and counsellor, EBR = European Board of Radiology, ED = European Diploma, ER = emergency radiology, ESR = European Society of Radiology, ETC = European Training Curriculum, LO = learning objective, QM = quality management, NL = non-adjustable limitations

**Table 2:**
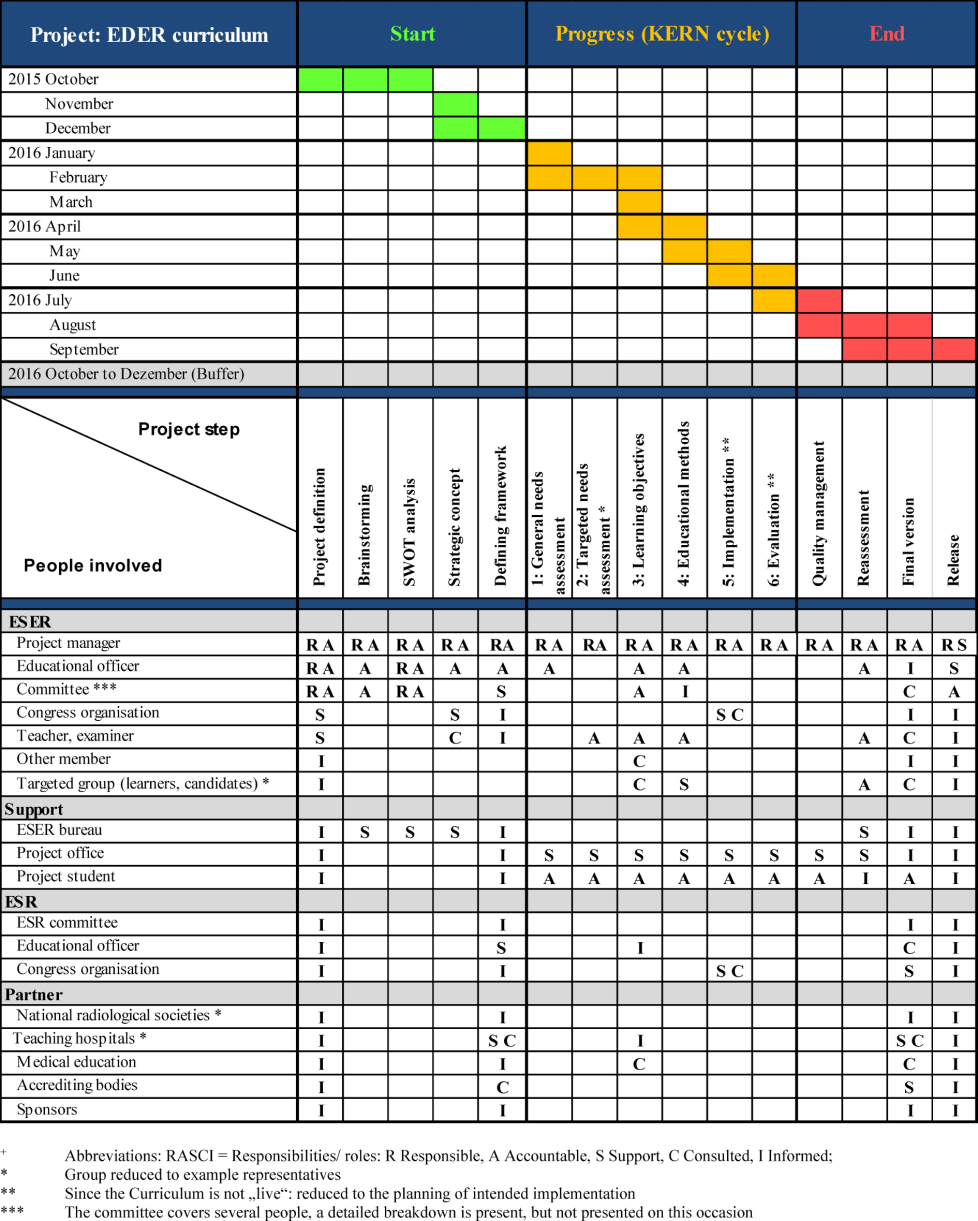
EDER project summary with time schedule (GANTT, on top) and responsibilities (RASCI+, at the bottom).

**Table 3:**
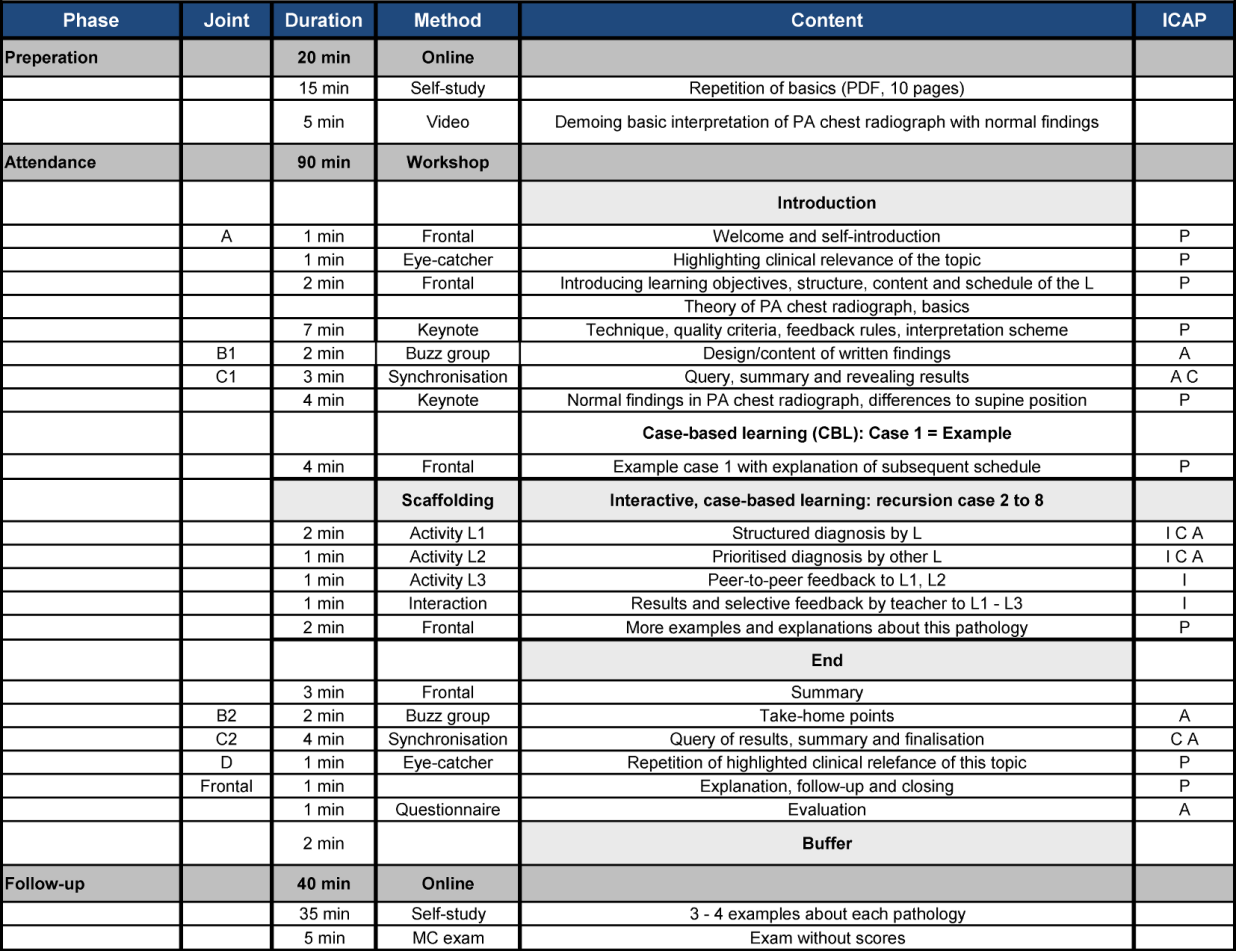
Schedule of a 90-minute workshop (attendance) using the example of thoracic imaging interpretation, which includes a decentralized preparation and follow-up (also see Fig. 3 and Tab. 4). Abbreviations: L = learner (Number to highlight different people), A = introduction, B = group phase, C = synchronisation of groups, D = ending; ICAP = activity level of L; I: interactive, C: constructive, A: active, P: passive.

**Table 4:**
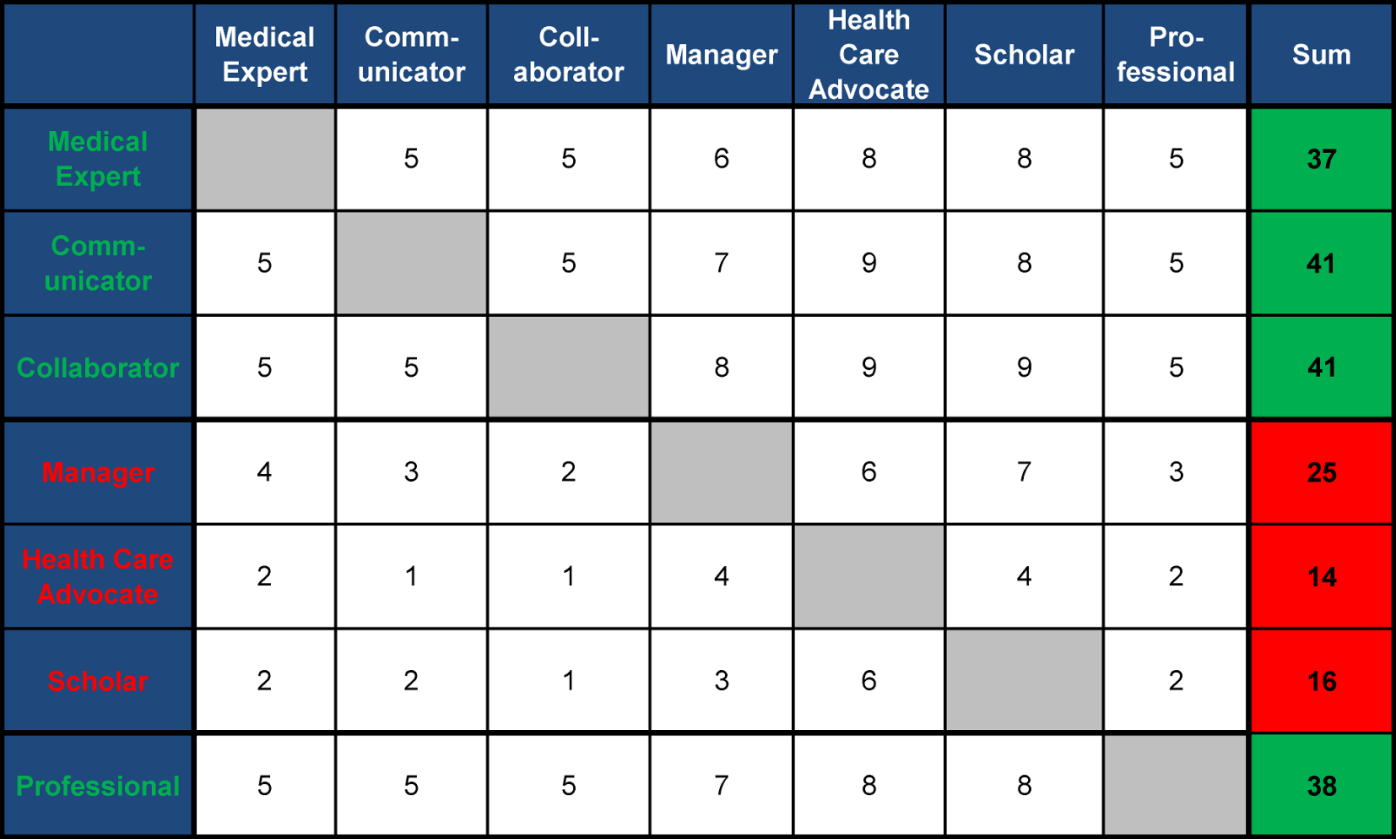
Principle of matrix analysis* using the example of determining the value of different CanMEDS roles for the workshop “Polytrauma” (Topic 9). * The matrix analysis is used for determining value. Above the main diagonal, the value of “row” versus “column” is rated per cell with a score of 1 to 10 (higher score means more value, 5 means equal value). Scores below the main diagonal result from mirroring, so that the sum of the mirrored cell results in the maximal score (in this case, 10). The example showed that the communicator, collaborator, and professional and medical expert roles are important, the manager role is intermediate, and the scholar and health advocate roles were rated as less important. This can be used directly for planning a course and should be provided as a tool for teachers.
